# The Two-Component Adjuvant IC31® Boosts Type I Interferon Production of Human Monocyte-Derived Dendritic Cells via Ligation of Endosomal TLRs

**DOI:** 10.1371/journal.pone.0055264

**Published:** 2013-02-06

**Authors:** Attila Szabo, Peter Gogolak, Kitti Pazmandi, Katalin Kis-Toth, Karin Riedl, Benjamin Wizel, Karen Lingnau, Attila Bacsi, Bence Rethi, Eva Rajnavolgyi

**Affiliations:** 1 Department of Immunology, Medical and Health Science Centre, University of Debrecen, Debrecen, Hungary; 2 Department of Rheumatology, Beth Israel Deaconess Medical Center, Harvard University Medical School Boston, Massachusetts, United States of America; 3 Intercell AG, Vienna, Austria; 4 Department of Microbiology, Tumor and Cell Biology, Karolinska Institute, Stockholm, Sweden; University of Pittsburgh, United States of America

## Abstract

The aim of this study was to characterize and identify the mode of action of IC31®, a two-component vaccine adjuvant. We found that IC31® was accumulated in human peripheral blood monocytes, MHC class II positive cells and monocyte-derived DCs (moDCs) but not in plasmacytoid DCs (pDCs). In the presence of IC31® the differentiation of inflammatory CD1a^+^ moDCs and the secretion of chemokines, TNF-α and IL-6 cytokines was inhibited but the production of IFNβ was increased. Sustained addition of IC31® to differentiating moDCs interfered with IκBα phosphorylation, while the level of phospho-IRF3 increased. We also showed that both IC31® and its KLK component exhibited a booster effect on type I IFN responses induced by the specific ligands of TLR3 or TLR7/8, whereas TLR9 ligand induces type I IFN production only in the presence of IC31® or ODN1. Furthermore, long term incubation of moDCs with IC31® caused significantly higher expression of IRF and IFN genes than a single 24 hr treatment. The adjuvant activity of IC31® on the IFN response was shown to be exerted through TLRs residing in the vesicular compartment of moDCs. Based on these results IC31® was identified as a moDC modulatory adjuvant that sets the balance of the NF-κB and IRF3 mediated signaling pathways to the production of IFNβ. Thus IC31® is emerging as a potent adjuvant to increase immune responses against intracellular pathogens and cancer in future vaccination strategies.

## Introduction

Protein antigens of subunit vaccines are usually poorly immunogenic and require vaccine adjuvants to enhance their immunogenicity [1]. Most adjuvants have been shown to induce and/or enhance adaptive immune responses; however, the primary targets of adjuvants are not B- and T-lymphocytes but various cells types of innate immunity [2].

Based on their complex functional activities they have been identified as major coordinators of innate and adaptive immunity [3]. These cells can induce antigen-specific inflammatory T cell responses or, alternatively, suppress T cell activation or trigger regulatory T-lymphocyte responses thereby playing a pivotal role in the maintenance of peripheral tolerance [4]. DCs are generated from bone marrow precursors and represent a minor and remarkably heterogeneous population of cells [5]. Phenotypic, migratory and functional properties of DCs classify these cells to conventional DCs (cDCs) and plasmacytoid DCs (pDCs) [6]. cDCs derive from common precursors of monocytes and macrophages and generate both lymphoid organ resident as well as inflammatory subsets, which can be activated by various endogenous and exogenous stimuli [7], [8]. In contrast to activated cDCs that migrate from peripheral tissues to draining lymph nodes through lymphatics, pDCs reside in peripheral lymphoid organs and when activated migrate to inflamed tissues. Monocyte-derived DCs (moDCs) with inflammatory properties have recently been validated as functionally competent cDCs, which can be generated *in vivo* under inflammatory conditions [9] and *in vitro* in the presence of specific cytokines [10]. moDCs continuously sense, internalize and process protein antigens and present their degradation products to naive T-lymphocytes with concomitant co-stimulatory signals that drive T-cell activation and differentiation to effector cells with different functional attributes [11].

Due to the expression of a wide variety of pattern recognition receptors (PRRs) specific for different phylogenetically conserved pathogen-associated molecular patterns (PAMPs) cDCs are primary targets of both microbial and particulate adjuvants [1]. Specific ligands of PRRs, liposomes and microparticles activate DCs directly, whereas mineral salts and various antigen delivery systems exert indirect effects on DCs through the stimulation and recruitment of other blood [12] or stromal cells [13]. It is also well established that the combination of multiple stimulatory signals boost adjuvant effects [14], [15] and induces the accumulation of inflammatory cells at the injection site [16]. However, the molecular mechanisms mediating these complex effects are still poorly characterized [12].

IC31® is a two-component adjuvant consisting of the artificial antimicrobial cationic peptide KLK acting as a vehicle [17] and the TLR9-stimulatory oligodeoxynucleotide ODN1a [18], [19]. Several *in vivo* studies in murine model systems revealed the Th1 and/or Th17 polarizing effect of IC31® when used as an adjuvant in combination with mycobacterial antigens [20]–[22], and the efficacy of IC31® in anti-mycobacterial vaccination of healthy volunteers was also shown [23], [24]. The IC31® component KLK was shown to facilitate the uptake and delivery of ODN1a into TLR9-positive intracellular vesicular compartments of human moDCs [25], [26] and based on these properties IC31® was implicated to have a profound effect on immune responses triggered by TLR9-agonists [27]. TLR9-mediated stimulation is intimately linked to type I interferon production and previous studies in a mouse model revealed the capability of IC31® to induce peptide specific cytotoxic T cells (CTL) in a type I interferon and Stat1 dependent manner, however, the exact mechanism was not identified [28], [29]. Based on these results we hypothesized that type I interferon production contributed to the adjuvant potential of IC31® and we sought to analyze the effects of IC31® in human DCs, where TLR9 expression and TLR9-mediated type I IFN induction has been associated to pDCs [30], whereas the role of TLR9 in cDCs has remained controversial [31].

The major goal of the present study was to understand how IC31® regulates human pDCs and cDCs by monitoring its uptake by the different cell types and the effect IC31® exerts on DC differentiation and on various activation pathways. We found that IC31® does not modulate pDC functions but it has profound effects on moDC differentiation and activation. Type I IFN production was stimulated by IC31® enhancing IRF3 activation, whereas it inhibited the NF-κB mediated chemokine and pro-inflammatory cytokine responses. Furthermore IC31® exerts strong booster effect on the IFNβ production of moDCs activated through vesicular TLRs.

## Materials and Methods

### Isolation and Culturing of Primary pDCs

Peripheral blood mononuclear cells (PBMC) were isolated by Ficoll-Paque (GE Healthcare, Uppsala, Sweden) density gradient centrifugation of heparinized leukocyte-enriched buffy-coats of healthy donors drawn at the Regional Blood Center of Hungarian National Blood Transfusion Service (Debrecen, Hungary) in accordance with the written approval of the Director of the National Blood Transfusion Service and the Regional and Institutional Ethics Committee of the University of Debrecen, Medical and Health Science Centre (Debrecen, Hungary). Written informed consent was obtained from the donors prior blood donation and their data were processed and stored according to the directives of the European Union. Plasmacytoid DCs were purified from PBMC by negative selection using the immunomagnetic cell separation kit (Miltenyi Biotec, Bergish Gladbach, Germany) according to the manufacturer’s instruction. After separation on VarioMACS magnet the purity of BDCA2^+^ BDCA4^+^ CD123^+^ pDCs was >98%, as confirmed by flow cytometry.

Freshly isolated pDCs were seeded at 1x10^5^ cells/well in 96-well flat-bottom plates in RPMI 1640 medium (Sigma-Aldrich, St. Louis, MO, USA) supplemented with 2 mM L-glutamine (Sigma-Aldrich), 100 U/ml penicillin, 100 ng/ml streptomycin, 10% heat-inactivated FCS (Invitrogen, Carlsbad, CA, USA) and 50 ng/ml recombinant human IL-3 (Peprotech EC, London, UK).

### Stimulation and Flow Cytometric Analysis of pDCs

Freshly isolated primary pDCs were treated with 10 nM KLK and 0.4 nM ODN1a by using the two components separately or mixed according to the IC31® mixing protocol provided by Intercell AG. The treated cells were incubated at 37°C in 5% CO_2_ humidified atmosphere for 24 h and then the supernatants were collected and stored at −70°C until cytokine measurements, whereas the cell were analyzed by flow cytometry. For stimulation of pDCs in whole blood, peripheral blood samples of healthy donors were exposed to IC31® and its components for 24 h. After incubation the blood samples were labeled with fluorescence- conjugated monoclonal antibodies (mAbs) and were lysed with BD FACS Lysing Solution (BD Biosciences) to eliminate red blood cells before flow cytometric analysis. Expression of cell surface proteins was analyzed by staining the cells with FITC-labeled mAbs specific for CD40, CD62L (BD Pharmingen, San Diego, CA, USA), CD80 (BioLegend, Uithoorn, The Netherlands) and PE-labeled human mAbs recognizing CXCR4, CCR2, CCR7 (R&D System, Minneapolis, MN, USA), CD84 (BD Pharmingen) and APC-labeled human mAbs against CXCR3, CCR5, CD38 (R&D System) and PE-Cy5-labeled anti-CD83 (BD Pharmingen). Isotype-matched control antibodies (Abs) were obtained from BD Pharmingen. In whole blood samples pDCs were identified by single positive staining with the anti-BDCA-4 mAb conjugated with APC (Miltenyi Biotec) and was further identified by back gating to light scatter parameters to get a homogenous population of pDCs [32]. Fluorescence intensities were measured by FACSCalibur flow cytometer (BD Biosciences Immunocytometry Systems, Franklin Lakes, NJ, USA) and data analysis was performed by the FlowJo software (TreeStar, Ashland, OR, USA). Uptake of FITC-conjugated KLK and/or Cy5-labeled ODN1a by pDCs was investigated in PBMC cultures at both 37°C and 0°C. pDCs were identified in PBMCs by using PE-labeled human anti-BDCA-2 mAb (Miltenyi Biotec) and light scatter parameters.

### Isolation of Monocytes, Differentiation and Activation of moDCs and their Characterization by Flow Cytometry

Leukocyte-enriched buffy coats were obtained from healthy blood donors drawn at the Regional Blood Center of Hungarian National Blood Transfusion Service (Debrecen, Hungary). PBMCs were separated by standard density gradient centrifugation with Ficoll-Paque Plus (Amersham Biosciences, Uppsala, Sweden). Monocytes were purified from PBMCs by positive selection using immunomagnetic cell separation with anti-CD14 microbeads according to the manufacturer’s instruction (Miltenyi Biotec). After separation on a VarioMACS magnet 96–99% of the cells were CD14^+^ monocytes as measured by flow cytometry. Monocytes were cultured in 12-well tissue culture plates at a density of 2×10^6^ cells/ml in AIM-V medium (Invitrogen) supplemented with 80 ng/ml GM-CSF (Gentaur Molecular Products, Brussels, Belgium) and 100 ng/ml IL-4 (Peprotech EC). On day 2 the same amounts of GM-CSF and IL-4 were added to the cell cultures.

Phenotyping of resting and activated moDCs was performed by flow cytometry using anti-CD83 and anti-CD1a mAbs and isotype-matched control Abs (BD Pharmingen). Fluorescence intensities were measured by FACSCalibur cytometer (BD Biosciences) and data were analyzed by the FlowJo software (Tree Star). KLK-FITC and ODN1a-Cy5 uptake was investigated at 37°C and 0°C as control. The cell population of interest was gated according the forward and side light scatter properties. The possible cytotoxic effect of KLK, ODN1a and IC31® was monitored by an Annexin-V/PI kit.

Bacterial lipopolysaccharide (LPS) (Sigma-Aldrich), human recombinant IFNγ (Peprotech EC), high molecular weight polyinosinic:polycytidylic acid (pI:C), CL075 and CpG2216 (InvivoGen, San Diego, CA, USA) were used at concentrations indicated in the Figure legends. To prepare cell lysates for Western blotting DCs were activated for 24h. Cell culture supernatants for ELISA were collected and cell lysates were prepared for real-time quantitative polymerase chain reaction (QPCR) after 18–24-h stimulation. KLK and ODN1a were used as described above.

### RNA Isolation, cDNA Synthesis and QPCR

To analyze the relative changes in gene expression QPCR was performed as described previously [33]. Briefly, total RNA was isolated by TRIzol reagent (Invitrogen) and 1.5–2 μg of total RNA were reverse transcribed using SuperScript II RNase H reverse transcriptase (Invitrogen) and Oligo(dT)15 primers (Promega, Madison, WI, USA). Gene-specific TaqMan assays (Applied Biosystems, Foster City, CA) were used to perform QPCR in a final volume of 25 μl in triplicates using AmpliTaq DNA polymerase and ABI Prism 7900HT real-time PCR instrument (Applied Biosystems). Amplification of 36B4 was used as normalizing control. Cycle threshold values (Ct) were determined using the SDS 2.1 software. Constant threshold values were set for each gene throughout the study. The sequence of the primers and probes are available upon request.

### Chemokine Array

Chemokine gene expression profiling of control and IC31®-treated moDCs was studied with the Human Chemokines & Receptors PCR Array assay (SABiosciences, Frederick, MD, USA) in accordance with the manufacturer’s recommendations. Briefly, total RNA was isolated from the untreated and treated cells using RNeasy Mini Kit. After DNase I digestion first strand cDNA synthesis was performed by using the RT2 First Strand Kit (SABiosciences) following the manufacturer’s instructions and including a genomic DNA elimination step and external RNA controls. For each sample 900 ng of total RNA was reverse transcribed. Real-time PCR measurement was performed using the RT2 qPCR Master mix (SABiosciences) according to the manufacturer’s instructions on the ABI Prism 7000 real-time PCR platform. For each analysis 25 µl of the experimental cocktail of the cDNA samples and RT2 qPCR Master mix was distributed across the PCR array 96 well plates each of which contained 84 wells with a real-time PCR assay for different chemokine genes, 5 wells with assays for different housekeeping genes, a genomic DNA control, 3 replicate Reverse Transcription controls and 3 replicate Positive PCR controls. A two-step cycling program was used consisting of an initial activating step of 10 min at 95°C and a second step of 40 cycles of denaturation (95°C, 15 s) and annealing (60°C, 1 min). Finally, each plate underwent a melting curve program (95°C, 1 min; 65°C, 2 min (optics off); 65°C to 95°C at 2°C/min (optics on). Gene expression levels were determined as the inverse of the Ct value. Ct values greater than 35 or those not detected were set to 35. After cycling with real-time PCR the amplification data were analyzed and statistical significance was calculated by SABiosciences on-line software.

### siRNA Experiments

Gene specific siRNA knockdown was performed by SilencerSelect siRNA (Applied Biosystems) transfection on day 3 using GenePulser Xcell instrument (Bio-Rad, Hercules, CA, USA). Silencing of RLR genes was performed by RIG-I and MDA5 siRNA mix and Silencer Negative Control non-targeting siRNA (Applied Biosystems) was used as a negative control. The efficiency of siRNA treatments was tested before and after DC activation on days 5 and 6 by Q-PCR and Western blotting.

### Western Blotting

Cells were lysed in Laemmli buffer and the protein extracts were tested by Abs specific for TLR3 (Abcam, Cambridge, UK), MDA5 (Lifespan, Seattle, WA, USA), RIG-I, IκB-α, phospho-IκB-α, IRF3, phospho-IRF3 (Cell Signaling, Danvers, MA, USA) and β-actin (Sigma-Aldrich) diluted to 1∶1000, the secondary Ab was used at 1∶5000. Anti-rabbit or anti-mouse (pIκB-α) Abs conjugated to horseradish peroxidase (GE Healthcare) were used as secondary Abs. The SuperSignal enhanced chemiluminescence system was used for probing the target proteins (Thermo Scientific, Rockford, IL, USA). After the membranes had been probed for the target protein they were stripped and re-probed for β-actin.

### Cytokine Measurements

Culture supernatants of DCs were harvested 24 h after activation and the concentrations of IL-6, IL-8 and TNF-α were measured using OptEIA kits (BD Biosciences). The level of secreted IFNβ was measured by Human Interferon beta ELISA Kits (Cell Sciences, Canton, MA, USA) according to the manufacturer’s protocol. Absorbance measurements were performed with a Synergy HT reader (Bio-Tek Instruments, Winooski, VT, USA) at 450 nm. Secreted MIP-1α, MIP-1β and IFN-α levels were measured with FlowCytomix kits (eBioscience, Vienna, Austria) by flow cytometry. The data were analysed by the FlowCytomix Pro 2.2 Software (eBioscience).

### Statistics

Data are presented as mean ± SEM, p values were calculated using the Student’s *t* test. p<0.05 was considered as statistically significant; *p<0.05, **p<0.01, ***p<0.005 and ****p<0.001.

## Results

### 1. Accumulation of IC31® and its Components in Human Peripheral Blood Mononuclear Cell Populations

Cell type-specificity and cellular compartmentalization of PRRs have an important impact on the functional activity of vaccine adjuvants [34]. Thus we first tried to identify the cell types in human PBMC preferentially involved in the accumulation of IC31® by using the fluorescence labeled components, KLK and ODN1a of the adjuvant. We hypothesized that IC31® targets preferentially phagocytes and professional APCs. Taken the unique functional attributes of pDCs in type I IFN responses and the expression of TLR9 in this cell type, we tested the effects of IC31® on the phenotype and functional activities of pDCs in whole blood and in primary pDC cultures. We found that fluorescence intensities of blood cells were increased in the presence of IC31® and its KLK and ODN1a components in a temperature independent manner but temperature dependent active internalization of the labeled components could not be observed (Figure S1). When pDCs were tested in erythrocyte depleted peripheral blood IC31® could not induce the expression of the CD80, CD83, CD84, CD40, CD38 activation markers and CD62L expression on the surface (Table S1A). In line with the lack of pDC activation the cells were unable to produce IFNα, TNF-α, IL-6 and IL-8 cytokines (data not shown) and changes in the cell surface expression of CCR2, CCR5 and CXCR3 also could not be detected (Table S1B). However, we found significantly increased expression of CCR7 and CXCR4 on pDCs induced by IC31®. To test whether upregulation of these chemokine receptors was a direct effect of IC31® on pDCs we performed experiments with isolated pDCs after short term *in vitro* cultures, but no effect could be detected (Table S1B). These results suggested an indirect effect of IC31® on chemokine receptor expression likely mediated by other blood cell types and/or their products.

**Figure 1:**
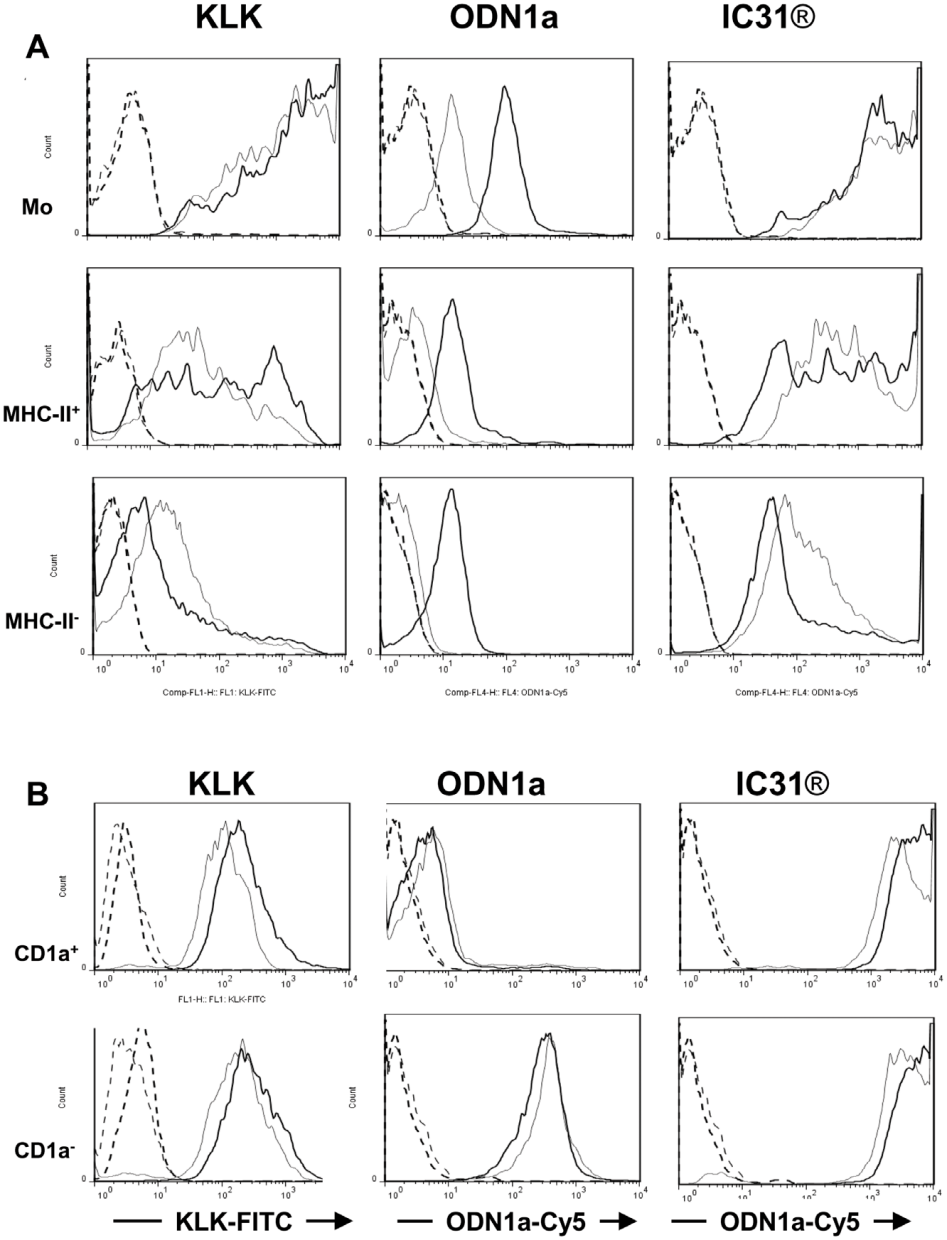
Distribution of fluorescent ODN1a, KLK and IC31® in human peripheral blood mononuclear cells and in monocyte-derived dendritic cell subsets. A - Human peripheral blood mononuclear cells (PBMC) were incubated with Cy5-conjugated ODN1a, FITC-conjugated KLK and IC31® containing both fluorescent components for 1 h either on ice (thin line) or at 37°C (thick line). Fluorescence intensities were measured by flow cytometry in monocytes (upper panel) identified by their light scatter properties, MHC class II^+^ (middle panel) and MHC class II^−^ (lower panel) lymphocyte populations discriminated by PE-conjugated HLA-D-specific Ab. Unstained controls are shown as thick (37°C) or thin (0°C) dashed lines. Cells stained by KLK-FITC (left column) or ODN1a-Cy5 (middle and right columns) are shown. A typical experiment out of three is documented. B - Human monocyte-derived DCs were harvested on day 5 of *in vitro* differentiation and incubated with KLK, ODN1a or IC31® as described in Figure 1A. The cells were kept either on ice (thin lines) or incubated at 37°C (thick lines). KLK-FITC (left column) and the ODN1a-Cy5 fluorescence intensities (middle and right columns) were evaluated by flow cytometry. CD1a positive (upper panel) and CD1a negative (lower panel) moDCs were discriminated by gating the cells based on their staining with a PE-conjugated CD1a-specific antibody. Controls, incubated without KLK-FITC or/and ODN1a-Cy5 are shown by thick and thin dashed lines (CD1a positive and CD1a negative moDCs, respectively). A typical experiment out of three is shown.

When we compared fluorescence intensities of different cell fractions of human PBMC we found that monocytes and MHC class II positive cells, which involve mostly B-lymphocytes, were stained very strongly by KLK-FITC as compared to MHC class II negative cells (Figure 1A, left column; Table 1). The highest fluorescence intensity was measured in monocytes (Figure 1A, left upper panel; Table 1) whereas staining of the MHC class II positive cells indicated a heterogeneous cell population with different fluorescence intensities (Figure 1A, left middle panel). The left column also indicates the temperature independent staining of the cells with KLK-FITC in contrast to ODN1a-Cy5, which exhibits a temperature dependent accumulation in all tested cell fractions (Figure 1A, middle column; Table 1). However, staining of ODN1a was shifted to higher fluorescence intensities when present in IC31® and its temperature dependency could not be detected anymore (Figure 1A right column; Table 1) indicating that KLK renders the cells capable to interact with ODN1a in a temperature independent manner. Although IC31® is accumulated by MHC class II positive cells, which also involve circulating DCs at low numbers, IC31® is most efficiently accumulated by monocytes that can give rise to moDCs in both peripheral and lymphoid tissues. Based on these results we performed our further experiments with moDCs and tested the effects of IC31® on cell differentiation, activation and functional activities.

**Table 1:** Distribution of IC31® and its components in peripheral blood mononuclear cells.

	ΔMFI values					
Treatment	KLK		ODN1a		IC31®	
Fluorescence	KLK-FITC		ODN1a-Cy5			
Temperature	37°C	0°C	37°C	0°C	37°C	0°C
monocytes	2066.55	1615.52	91.71	9.75	9907.81	9907.75
HLA-D^+^ lymphocytes	80.99	44.49	12.29	1.29	983.19	564.09
HLA-D^−^ lymphocytes	4.6	12.07	10.2	0.36	50	124.51

We have previously shown that based on the expression of CD1a, a protein presenting glyco- and phospholipids of pathogens such as *Mycobacterium tuberculosis*, two phenotypically and functionally distinct moDC subsets can be separated [33]. It was also shown that CD1a^−^ cells are more active in the internalization of exogenous compounds than their CD1a^+^ counterparts [33]. To control whether IC31® uptake was different in these moDC subsets we compared the fluorescence intensities in CD1a^+^ and CD1a^−^ cells at both 0°C and 37°C (Figure 1B). Marginal differences could be detected in fluorescence intensities measured in the CD1a^−^ and CD1a^+^ DC subsets suggesting the involvement of both cell types in interacting with IC31® and its components in a temperature independent manner.

### 2. Effect of Prolonged Presence of IC31® on Monocyte-derived Dendritic Cell Differentiation and Activation

Our previous studies revealed that CD1a^−^ and CD1a^+^ DCs are the result of consecutive differentiation steps and their ratio varies among individuals [33]. To control the long and short term effects of IC31® on moDCs we set up various *in vitro* experimental systems. In the first system (protocol A) moDCs were differentiated in the presence of IC31® and its components added to monocytes on day 0 and to differentiating moDC on day 2 (Figure S2A). In the second system (protocol B) a similar procedure was performed but extended by an additional IC31® treatment on day 5 together with the activation of moDCs by LPS plus IFNγ (LPS+IFNγ), or by various TLR ligands to mimic inflammatory conditions (Figure S2B). In the third system (protocol C) the IC31® adjuvant and its components were added on day 5 only, combined with the activation stimuli as in protocol B (Figure S2C).

**Figure 2:**
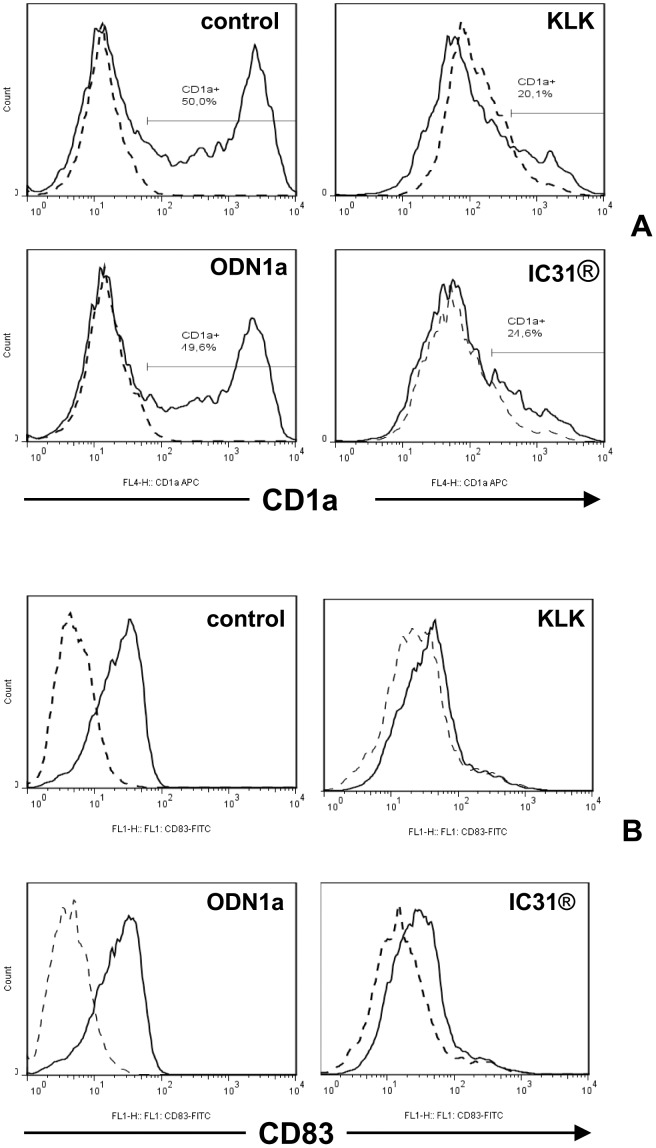
The effect of KLK, ODN1a and IC31® on the differentiation and activation of monocyte-derived dendritic cell subsets. A - Human moDCs were differentiated for 5 days in the absence (control) or presence of KLK, ODN1a or IC31®, added on days 0 and 2 to the cultures (Protocol A, Figure S2) together with the differentiating cytokines IL-4 and GM-CSF. The distribution of CD1a^−^ and CD1a^+^ cells was monitored by flow cytometry. Samples stained by CD1a-specific antibody or isotype-matched control antibody (dashed line) are shown. B – Human moDCs were differentiated as described in Figure 2A and on day 5 they were activated by 100 ng/ml LPS +10 ng/ml IFNγ in combination with KLK, ODN1a and IC31® for 24 h (Protocol B, Figure S2). Activation of DCs was evaluated on day 6 by detection of the cell surface expression of CD83 using flow cytometry. Data from a typical experiment out of three independent experiments are shown.

First monocytes were differentiated in the presence of IC31®, KLK or ODN1a according to Figure S2A and the ratio of CD1a^+^ and CD1a^−^ cells was monitored by flow cytometry. IC31® dramatically inhibited the generation of the CD1a^+^ cells and this effect could be attributed to KLK, as ODN1a had no effect (Figure 2A). Next we tested whether IC31® had any effect on the activation of moDCs induced by LPS+IFNγ on day 5 of culture by using protocol B (Figure S2B). Measuring cell surface expression of the activation molecule CD83 after 24 h we found that IC31® and its KLK component inhibited the activation of moDCs, while ODN1a had no effect (Figure 2B). Interestingly, the expression of CD86, present on both resting and activated moDCs was not modulated (data not shown). Based on these results we conclude that IC31® modulates moDC differentiation and activation and KLK has an essential role in these effects.

### 3. IC31® Modulates the Cytokine Profile of Human Monocyte-derived Dendritic Cells

**Figure 3:**
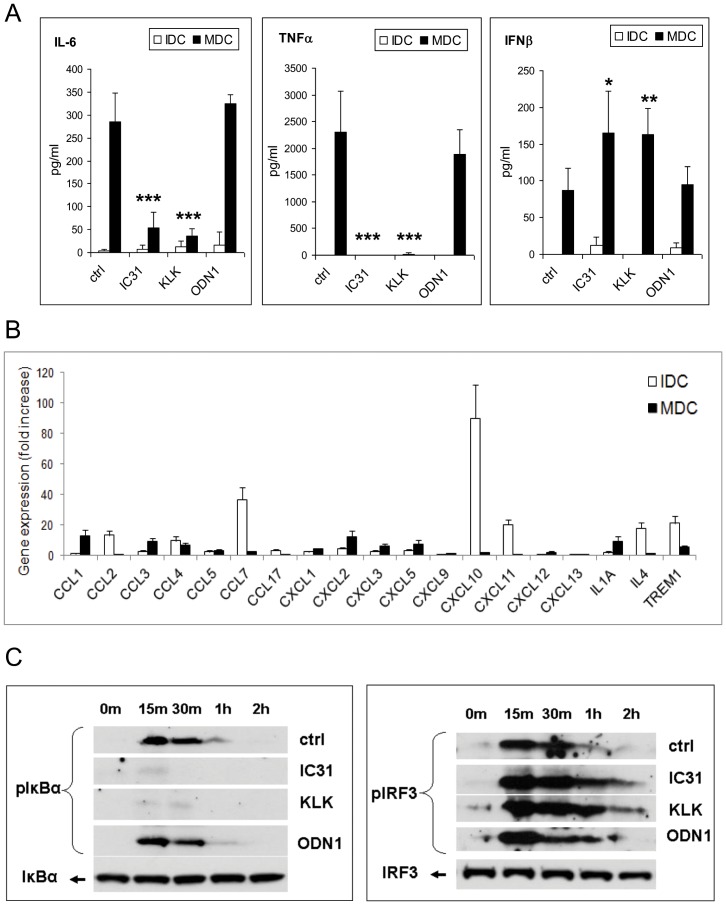
The effect of KLK, ODN1 and IC31® on the cytokine production of monocyte-derived dendritic cells. Human moDCs were differentiated and activated as described in Protocol B (Figure S2) (IDC: non-activated immature dendritic cells; MDC: 100 ng/ml LPS +10 ng/ml IFNγ -activated mature dendritic cells). A – Supernatants of DC cultures were collected on day 6 and the concentration of IL-6, TNF-α and IFNβ was measured by ELISA. Mean±SEM values of 3 independent experiments performed with moDCs obtained from different donors are presented. Statistically significant changes were calculated as described in the Material and Methods. B – Gene expression profile of chemokine and cytokine genes in DCs treated as described in Protocol B. Fold increase values of two independent donors are presented as mean±SD of triplicate measurements. C – Activation of the NF-κB and IRF3 pathways was evaluated in cell lysates by detection of phosphorylation state of IκBα and IRF3 using Western blotting. Data of a representative DC donor out of three are shown.

LPS and IFNγ are potent stimulators of human moDCs but as it is documented in the previous sections, IC31® has inhibitory effects on differentiation of the CD1a^+^ subset and on moDC activation monitored by CD83 expression. In line with this finding when cells were treated with LPS+IFNγ in combination with IC31® according to protocol B, secretion of TNF-α and IL-6 was significantly reduced (Figure 3A). Moreover, gene expression of the chemokines CCL2, CCL7, CXCL10, and CXCL11 was also inhibited as compared to resting moDCs (Figure 3B). Concomitant with these effects we also found statistically significant increase in IFNβ secretion from LPS+IFNγ-activated cells in the presence of IC31® or KLK, but not ODN1a (Figure 3A). To clarify the molecular background of these modulatory effects we sought to investigate the activation of NF-κB- and IRF3-mediated signaling pathways linked to chemokine, inflammatory cytokine and type I IFN producing cells, respectively (Figure 3C). Our results show that IC31® and KLK almost completely abolished IκBα phosphorylation while slightly increased and extended the phosphorylated state of IRF3, the transcription factor driving IFNβ1 gene expression. Thus IC31® is unable to activate the NF-κB pathway but rather supports the IRF3 mediated type I IFN response. As these signaling cascades mutually influence each other’s functional activities, this effect could be attributed either to the inhibition of IκBα activity or to the modulation of the balance of IκBα and IRF3 mediated pathways upon signaling flux redistribution [35]. These results also confirmed that this modulatory effect is attributed to KLK, the peptide component of IC31®.

### 4. Long Term Administration of IC31® Augments the Type I IFN Response of Monocyte Derived Dendritic Cells

**Figure 4:**
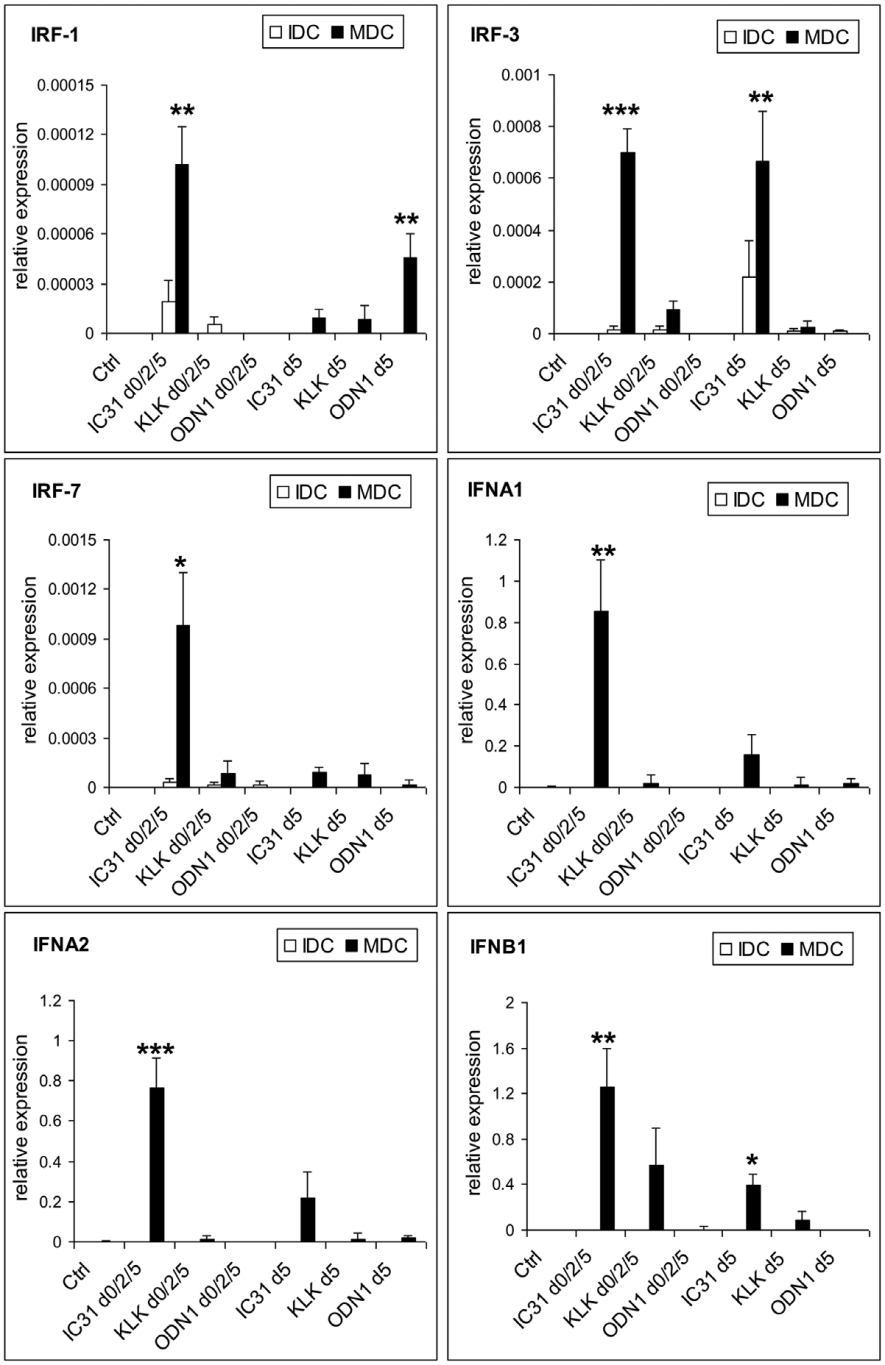
The effect of KLK, ODN1a and IC31® on the expression of IRF family transcription factors and type I IFNs. Human moDCs were differentiated for 5 days in the absence (control) or presence of KLK, ODN1a or IC31® added on day 0, 2 and 5 (Protocol B, Figure S2) to the cultures or on day 5 only (Protocol C). Relative expression of IRF1, IRF3 and IRF7 as well as IFNA1, IFNA2, IFNB1 genes was measured in resting (empty bars) and 100 ng/ml LPS +10 ng/ml IFNγ-activated (black bars) DCs by QPCR. Mean±SEM values of triplicate measurements were calculated from data of 3 independent donors.

Considering the remarkable increase in type I IFN secretion as a result of moDC activation in the presence of IC31® (Figure 3A) next we tested the effects of IC31® and its components on type I IFN pathway. In this system IC31® and its components were added on day 0, 2 and 5 (Figure S2B) as compared to a single treatment on day 5 (Figure S2C) combined with activation by LPS+IFNγ for 24 h and the expression profiles of selected members of the IRF and type I IFN family genes were measured in resting and activated moDCs by QPCR (Figure 4). IRF1 is a known enhancer of type I IFN signaling whereas IRF3 and IRF7 are master regulators of IFNA and IFNB1 expression [36]. We found that repeated administration of IC31® during moDC differentiation resulted in substantial enhancement of the expression of all IRFs and type I IFN genes as compared to using IC31® at day 5, only. These results clearly demonstrate that the prolonged presence of IC31® increases its efficacy. Furthermore, IRF3 gene expression was enhanced by either a single (day 5) or repeated (day 0, 2 and 5) administration of IC31® to the similar levels. These findings also suggest that if circulating monocytes acting as precursors of moDCs encounter IC31® it able to stimulate type I IFN production efficiently in developing moDCs, but it has a less prominent effect on already differentiated moDCs. These unique effects seem to be restricted to IC31® as it could be induced by the combination of KLK and ODN1a but not by its single components.

### 5. Mechanism of IC31® Action at the Level of Pattern Recognition Receptors Involved in Type I IFN Responses

**Figure 5:**
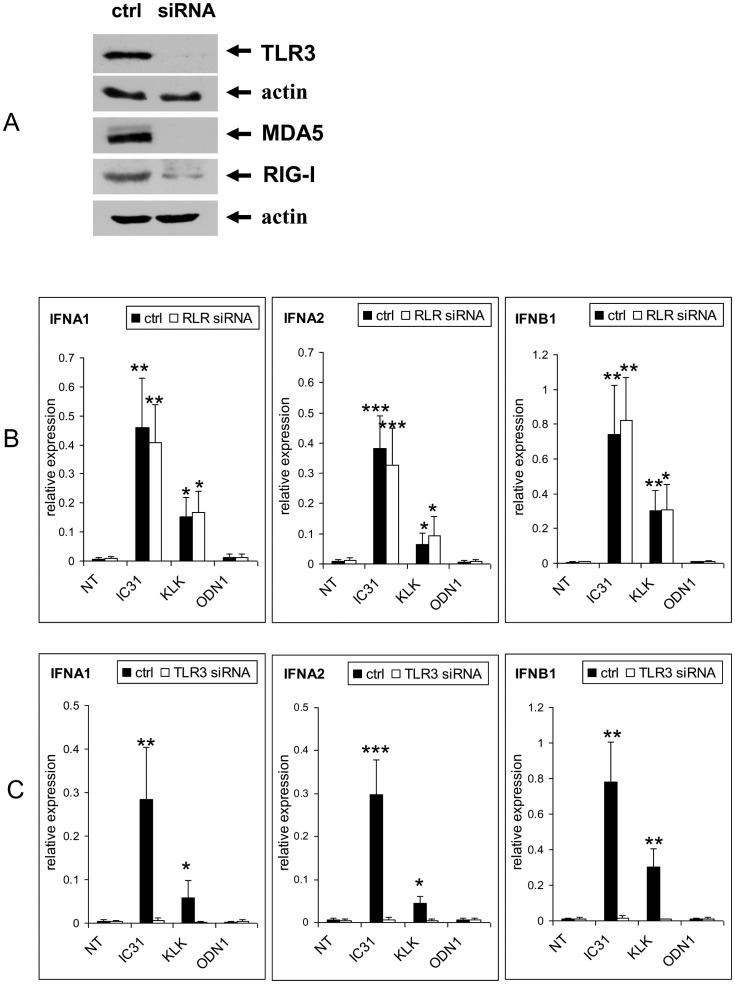
Identification of the receptors and signaling pathways involved in the enhancement of the type I IFN response by IC31®. Human moDCs were differentiated for 5 days in the absence (NT – “non-treated”) or presence of KLK, ODN1a or IC31® (Protocol B, Figure S2B). In Figures 5B, 5C, 5D and 5E moDCs were activated on day 5 by 20 μg/ml polyI:C for 24 hrs. In Figure 5F DCs were activated by 1 μg/ml CL075 and in Figure 5G by 5 μg/ml CpG2216 (IDC: non-activated immature dendritic cells; MDC: CL075 or CpG2216-activated mature dendritic cells). A – Validation of siRNA activity specific for TLR3 and RLR (RIG-I/MDA5) by Western blotting. B – Comparison of gene expression of type I IFN in control and RLR siRNA treated moDCs. Mean±SEM values of triplicates performed with DCs of two independent donors are shown. C – Comparison of gene expression of type I IFNs in control and TLR3 siRNA treated moDCs. Mean±SEM values of triplicates performed with moDCs of two independent donors are shown. D – Comparison of the levels of secreted IFNβ in control and RLR siRNA treated moDCs. Mean±SEM values of duplicates performed with moDCs of two independent donors are shown. E – Comparison of the levels of secreted IFNβ in control and TLR3 siRNA-treated moDCs. Mean±SEM values of duplicates performed with DCs of two independent donors are shown. F – Effect of TLR7/8 activation by CL075 on IC31®-, KLK- or ODN1a-treated moDCs monitored by IFNβ ELISA. Mean±SEM values of duplicates performed with DCs of two independent donors are shown. G – Effect of TLR9 activation by CpG2216 on IC31®-, KLK- or ODN1a-treated moDCs monitored by IFNβ ELISA. Mean±SEM values of duplicates performed with DCs of two independent donors are shown.

IFN responses can be mediated by various PRRs localized to intracellular vesicular compartments or cytosol. To assess the contribution of intracellular TLRs and cytosolic RIG-like receptors (RLRs) to type I IFN production in the context of IC31® treatment we studied the PRR ligand induced activation of moDCs in the absence or long term presence of IC31® and its components. To exclude the involvement of RLRs in IC31®-modulated production of IFNβ induced by polyI:C we performed siRNA experiments in which we silenced both RIG-I and MDA5 (Figure 5A). Our results revealed that the down-regulation of RLRs had no effect on the stimulatory effect of IC31® on the polyI:C induced IFNβ expression measured at both gene (Figure 5B) and protein levels (Figure 5D). Similar results were obtained for IFNα gene expression but in this case moDCs did not produce detectable levels of IFNα (data not shown). In contrast to these result silencing of the TLR3 gene by specific siRNA (Figure 5A) resulted in dramatic abrogation of IFNβ mRNA and cytokine levels (Figure 5C, E). These results confirm that the type I IFN enhancing activity of IC31® does not depend on RLR mediated IFNβ production in human moDCs (Figure 5B, D) but is mediated by vesicular TLR3 induced signaling. This led us to hypothesize that IC31® is able to interact with intracellular PRRs localized to the endo/lysosomal membrane.

It is well established that cDCs express a specific combination of TLRs that involve TLR3, TLR7, TLR8 and TLR9 however, the functional role of TLR9 in this cell type is still controversial. Previous studies demonstrated that in human moDCs IC31® co-localizes with TLR9 [25] but the functional significance of this compartmentalization has not been explored. To check the possible booster effect of IC31® on IFNβ production mediated by the candidate vesicular sensors we used CL075 and CpG2216 as specific ligands of TLR7/8 and TLR9, respectively. Our results showed that specific ligation of TLR7/8 by CL075 resulted in significantly increased IFNβ production, which was further enhanced upon repeated IC31® treatment according to protocol B (Figure 5F). Interestingly, CpG2216 stimulation of moDCs did not induce IFNβ production by itself, however, repeated pre-treatment of moDCs by IC31® or ODN1a (protocol B) resulted in moderate production of IFNβ cytokine (Figure 5G). Thus IC31® renders TLR9 functional in moDCs and this effect is attributed to its ODN1a component. As TLR9 gene expression was not affected by IC31® (data not shown) we propose that targeting IC31® or ODN1a to LAMP1^+^ endolysosomal acidic membrane vesicles [25] ensures the required pH-dependent translocation and sufficient concentration of the CpG2216 ligand to trigger TLR9 in human moDCs.

## Discussion

Most prophylactic vaccines are based on the capability to trigger protective antibody responses however, provoking potent cellular immune responses against intracellular pathogens, which often cause persistent infections, requires the priming and/or modulation of inflammatory T-lymphocytes by properly activated DCs (reviewed in [3]).

Despite their low numbers in the circulation pDCs are considered as highly potent APCs of viral antigens and upon activation they differentiate toward natural IFN producing cells. This prompted us to study the effect of IC31® on this cell type. The expression of cell surface proteins associated with the activation and migration of pDC were not influenced by the treatment of the cells with IC31® or its components. As the expression of CXCR4 and CCR7 chemokines, involved in lymph node homing of pDCs was increased only in the presence of other blood cells but not in isolated pDCs, this phenomenon was attributed to the indirect effect of other stimulated cells present in peripheral blood. In line with the lack of pDC activation and induction of chemokine and cytokine secretion these results supported the notion that pDCs are not the primary targets of the IC31® adjuvant. Thus we focused to analyzing the response of human monocytes and differentiating moDC to IC31® and monitored its effects on various PRR coupled signaling pathways. We found that i) IC31® was efficiently accumulated in human blood monocytes and moDCs; ii) in the presence of IC31® the generation of inflammatory CD1a^+^ DCs was inhibited and the surface expression of CD83 did not increase, but CD86 expression remained intact; iii) the expression of chemokines and the secretion of TNF-α and IL-6 cytokines decreased but IFNβ secretion increased; iv) long term presence of IC31® prevented or inhibited IκBα phosphorylation while extended the phosphorylated state of IRF3; v) IC31® exhibited a booster effect on ligand induced vesicular TLR-mediated induction of IFNβ secretion.

IC31® has extensively been characterized in terms of its adjuvant effects [20]–[24], intracellular localization [26] and its role in TLR9 dependent activation of moDCs [28]. However, understanding of its mode of action and its effects on PRR triggered signaling cascades in human moDCs has not been analyzed so far. To assess the short and long term *in vitro* effects of IC31 on moDC activation and differentiation we set up different treatment protocols. The experimental data revealed that IC31® and its KLK component inhibited the transition of CD1a^−^ cells to more differentiated CD1a^+^ moDCs and interfered with moDC activation induced by the TLR4 ligand LPS combined with IFNγ. As a functional consequence the treated cells were unable to exhibit the typical phenotypic and functional changes of moDC activation, as increased expressions of CD1a and CD83, however, CD86 expression was not affected. This semi-mature differentiation and activation state of moDCs was associated to decreased NF-kB activation and to the unusual capability of the treated cells to secrete high amounts of IFNβ under the control of and in collaboration with IRFs. Thus IC31® could be identified as a moDC modulator that may have a profound effect on the balance of NF-kB and IRF3 mediated signaling pathways. This effect was also verified by a gene array showing complete inhibition of the mRNA expression of several CCL and CXCL chemokine genes known to be under the control of NF-κB and involved in the recruitment of monocytes. In our previous work we have shown that the intracellular sensors RIG-I and MDA5 in human moDCs are expressed in a DC subset-specific manner and are able to trigger the IRF3 mediated IFNβ pathway independently of NF-κB activation [37] supporting the notion that their collaboration or independent action relies on a delicate balance that can be harnessed for the development of potent but non inflammatory vaccine adjuvants.

To further analyze the interplay of the type I IFN coupled pathway with NF-κB mediated signaling we tested the involvement of other IFN response-related genes and proteins. We could demonstrate that IC31® and with lower extent KLK increase type I IFN responses induced by the specific ligands of TLR3 or TLR7/8. As these TLRs could be further stimulated for type I IFN production upon receptor ligation, this modulatory effect could be attributed in part to KLK, which facilitates the transport and accumulation of the specific TLR ligands into endosomal compartments, where they stimulate the resident TLRs. Although previous results demonstrated that TLRs localized to the same vesicular compartment may interfere with each other’s functional activities and may exert inhibitory effects [38], [39], our results point to synergistic action of IC31® and IFN signaling. Indeed, CpG2216 itself did not trigger type I IFN release from moDC, whereas co-administration of the TLR9 ligand with IC31® or ODN1 led to notable production of IFNβ.

**Figure 6:**
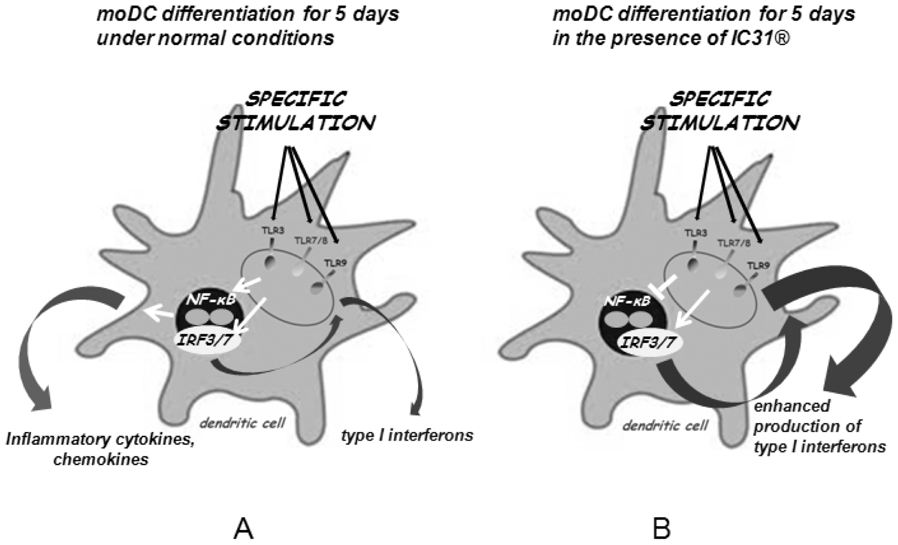
Proposed mode of action of IC31®. A – After specific ligation of the endosomal TLRs, moDCs produce inflammatory cytokines and type I interferons as a result of NF-κB and IRF3/7 signaling, respectively. B – When moDCs are differentiated in the presence of IC31®, they exhibit altered cytokine profile that is characterized by suppressed production of inflammatory cytokines and significantly increased levels of IFNβ.

We propose that the adjuvant effects of IC31® rely on the vesicular targeting of TLR ligands by KLK and the ODN1a-induced triggering of TLR9 for initial type I IFN production. This baseline effect can be further enhanced by additional TLR-specific ligands that known to have the potential to induce vesicular maturation essential for DC activation and potent antigen presentation. IC31® thus acts as a signaling pathway-restricted adjuvant. It is also important to note that the spatial distribution of IC31® may be of high importance concerning its adjuvants effects as long-term administration of IC31® resulted in significantly higher expression of IRF and IFN genes as compared to a single 24 h treatment, only. We hypothesize that repeated addition of IC31® leads to the gradual accumulation of the adjuvant in the endosomal membranes that may contribute to this booster effect (Figure 6).

Besides increasing the magnitude of adaptive immune responses, adjuvants also play an instructive role in driving immune responses to the most appropriate directions to confer protection against the target pathogen [40]. Although moDCs treated with IC31® did not express CD1a and CD83, they kept their expression of the costimulatory molecule CD86, which may be sufficient for the initiation and sustainment of a proper T cell response. We suggest, that together with the significantly increased levels of secreted IFN-β, this may lead to a Th1-polarized immune response. Efficient protection against intracellular pathogens requires the activation of CD4^+^ helper T-cells polarized to Th1 type adaptive immune responses and stimulation of DCs to induce potent and durable activation of CD8^+^ T-cells. These requirements can be achieved by agonists of TLR3, TLR4, TLR7/8 and TLR9 facilitating the access of the antigen to the MHC class I processing pathway and inducing type I IFN responses [1]. TLR activation in the same DC presenting the antigen is a crucial requirement of efficient CD4^+^ T cell activation and Th1 polarization, which also demonstrates the importance of cytokines produced by antigen carrying APCs [41], [42]. Our results clearly show that moDCs produce increased level of IFNβ when IC31® is co-administered with endosomal TLR-ligands. This augmented type I IFN production may potentiate and enhance the adjuvant effect of IC31® on the adaptive immune response. Furthermore, besides its antiviral activity, IFNβ has also been shown to act as a strong anticancer factor [43], [44]. Thus, we propose that IC31® is an effective adjuvant acting through the endosomal TLR system of moDCs and represents a potent tool for future vaccination strategies against intracellular pathogens and cancer.

## Supporting Information

Figure S1 Characterization of the effect of IC31® and its components on pDCs. Treatment and phenotyping of pDCs were performed as described in Materials and Methods. pDCs were incubated with Cy5-conjugated ODN1a, FITC-conjugated KLK and IC31® containing both fluorescent components for 1 hr either on ice (0°C) or at 37°C. Fluorescence intensities were measured by flow cytometry. Unstained controls are shown as thin lines. A typical experiment out of four is documented.
(RAR)

Figure S2 Protocols for short-term and long-term treatments of cDCs. Figure S2A represents a system in which moDCs were differentiated in the presence of IC31® that was added to monocytes on day 0 and to differentiating moDC on day 2 generating 5-day resting DCs (Figure S2A). In the second system a similar procedure was performed extended by an additional adjuvant treatment on day 5 together with the activation of DCs by exposure to LPS plus IFNγ (LPS+ IFNγ) or by various TLR ligands to mimic inflammatory conditions (Figure S2B). According to the third protocol, the IC31® adjuvant was added on day 5 only, combined with the activation stimuli as in protocol B (Figure S2C).
(RAR)

Table S1 Fluorescence intensities of activated pDCs were measured as described in Material and Methods. A – Expressions of CD80, CD83, CD84, CD40, CD38 and CD62L on the surface of pDCs. B – Fluorescence intensities of pDCs measured in whole-blood and in *in vitro* cultured pDCs treated with IC31®, KLK and ODN1a. Mean±SEM data of 3–4 independent experiments performed with cells derived from independent donors are shown. Significant changes are presented in bold (*p<0.05, **p<0.01).
(RAR)
