# Correction: The Two-Component Adjuvant IC31® Boosts Type I Interferon Production of Human Monocyte-Derived Dendritic Cells via Ligation of Endosomal TLRs

**DOI:** 10.1371/annotation/322893f7-942c-4059-9295-7ecd26162aca

**Published:** 2013-10-24

**Authors:** Attila Szabo, Peter Gogolak, Kitti Pazmandi, Katalin Kis-Toth, Karin Riedl, Benjamin Wizel, Karen Lingnau, Attila Bacsi, Bence Rethi, Eva Rajnavolgyi

Figure 5D, Figure 5E, and Figure 5F) are missing. Please see the corrected Figure 5 here: http://www.plosone.org/corrections/pone.0055264.cn.g005.tif

